# Innovations in Meta-Analytic and Computational Methods in the Neuroscientific Investigation of Psychiatric and Neurological Disorders

**DOI:** 10.3390/brainsci15121323

**Published:** 2025-12-12

**Authors:** Chris H. Miller, Thomas J. Farrer, Jonathan D. Moore, Matthew J. Wright, Caitlin Baten, Ellen Woo, J. Paul Hamilton, Matthew D. Sacchet, Lance D. Erickson, Shawn D. Gale, Dawson W. Hedges

**Affiliations:** 1 Department of Psychology, California State University, Fresno, CA 93740, USA; mattwright@mail.fresnostate.edu (M.J.W.); caitlin.baten@uconn.edu (C.B.); ewoo@mail.fresnostate.edu (E.W.); 2 Idaho WWAMI Medical Education Program, University of Idaho, Moscow, ID 83844, USA; tfarrer@uidaho.edu (T.J.F.); jonathandm@uidaho.edu (J.D.M.); 3 Department of Psychiatry, University of California, San Francisco, CA 94158, USA; 4 Department of Psychological Sciences, University of Connecticut, Storrs, CT 06269, USA; 5 Department of Biological and Medical Psychology, University of Bergen, 5020 Bergen, Norway; paul.hamilton@uib.no; 6 Department of Psychiatry, Meditation Research Program, Harvard Medical School, Boston, MA 02115, USA; meditationadministration@mgh.harvard.edu; 7 Department of Psychiatry, Meditation Research Program, Massachusetts General Hospital, Boston, MA 02114, USA; 8 Department of Sociology, Brigham Young University, Provo, UT 84602, USA; lance_erickson@byu.edu; 9 Department of Psychology, Brigham Young University, Provo, UT 84602, USA; shawn_gale@byu.edu (S.D.G.); dawson_hedges@byu.edu (D.W.H.)

**Keywords:** meta-analyses, functional magnetic resonance imaging (fMRI), positron emission tomography (PET), neuroinflammation, genome-wide-association studies (GWASs), polygenic risk scores (PRSs), psychiatric disorders, neurological disorders

## Abstract

Recent advancements in neuroimaging and genetics have generated a rapid proliferation of primary studies in these fields, leading to the development and application of meta-analytic methods, which have contributed substantially to our understanding of psychiatric and neurological disorders. The current narrative review discusses four such innovations and applications in meta-analytic techniques and how they have advanced our understanding of clinical conditions: (1) multilevel kernel density analysis (MKDA) of functional magnetic resonance imaging (fMRI) studies, (2) meta-analyses of positron emission tomography (PET) imaging of neuroinflammation, (3) Enhancing Neuroimaging Genetics through Meta-Analysis (ENIGMA) Consortium neuroimaging protocols, and (4) meta-genome-wide association studies (Meta-GWASs) and polygenic risk scores (PRSs). These meta-analytic methods have contributed substantially to our understanding of psychiatric and neurological disorders by refining robust neural models, identifying transdiagnostic and disease-specific biomarkers of inflammation, uncovering numerous genetic risk variants with improved prediction models, and underscoring the polygenic and pleiotropic architecture of these conditions. Future research should continue to develop techniques for harmonizing multimodal data analysis, pursue both biomarker- and mechanism-driven approaches to discovery, and leverage biological discoveries to advance development of precision treatments and diagnostic frameworks.

## 1. Introduction

The widespread availability of functional neuroimaging and genomic methods and their practical applications in advancing our understanding of clinical disorders have led to a tremendous number as well as acceleration of publications [1,2]. This rapid proliferation of primary studies has necessitated the development of increasingly sophisticated methodologies, including *meta-analyses*, studies based on statistical techniques used to quantitatively synthesize findings from multiple independent primary studies, and *computational tools*, including programs and algorithms to construct and analyze brain maps and execute simulations.

In particular, meta-analytic techniques, which are often combined with computational tools, enable investigators to aggregate large bodies of literature, thereby substantially increasing statistical power, to address inconsistencies among primary studies, and to provide statistically robust conclusions [3,4]. By combining results from across studies, meta-analyses also enable researchers to expand the diversity of samples and increase heterogeneity in study design, thereby increasing generalizability of study findings. These strengths are particularly noteworthy given ongoing challenges with replication [5,6], which high-powered meta-analyses with diverse samples and heterogenous study designs play an important role in addressing. Further, meta-analyses of results from across primary studies can enable the identification of patterns in the data not readily observable in a single primary study. For example, disorders examined across individual primary studies can be systematically aggregated in a meta-analysis to identify transdiagnostic and disorder-specific patterns of neural activation [7].

The current review discusses four such innovations in meta-analytic techniques, including analysis of repository data, and how they have advanced our understanding of clinical disorders: (1) multilevel kernel density analysis (MKDA) of functional magnetic resonance imaging (fMRI) studies, (2) meta-analyses of positron emission tomography (PET) imaging of neuroinflammation, (3) Enhancing Neuroimaging Genetics through Meta-Analysis (ENIGMA) neuroimaging protocols, and (4) meta-genome-wide association studies (Meta-GWASs) and polygenic risk scores (PRSs).

## 2. Multilevel Kernel Density Analysis of Functional Magnetic Resonance Imaging Studies

### 2.1. Overview

MKDA [8] is a type of coordinate-based meta-analysis (CBMA) that is now well-established in whole-brain fMRI activation studies and that has been successfully applied in the study of psychiatric disorders [9,10,11]. MKDA enables investigators to identify statistically robust patterns of differential neural activation between comparison groups or within-group effects in a body of literature from selected primary studies.

### 2.2. Methodology

In particular, the MKDA process is conducted in three phases. First, *literature* *screening* is conducted by searching relevant literature databases such as PubMed comprehensively for primary studies, according to predefined inclusion criteria and in a manner consistent with PRISMA standards [12]. These inclusion criteria and the accompanying study protocol are often also preregistered with a database such as PROSPERO [13]. Second, *data extraction* involves obtaining the reported brain coordinates and samples sizes as well as other desired information from each primary study and organizing this information into a tracker file for analysis. Third, *data analysis*, the core process in MKDA, involves applying a series of algorithms, using custom scripts developed on Matlab and AFNI, that assign clusters, or kernels, to the extracted coordinates and build whole-brain indicator maps for each primary study and then a global contrast map for the meta-analysis, which can be registered to Talairach [14] or MNI [15] space.

Some of these algorithms involve applying two *thresholds* to determine whether a given voxel or cluster, respectively, is statistically significant. The first threshold, the *meta-analytic statistic threshold (MAST)*, reflects whether a given voxel is statistically significant and is computed as the weighted proportion of primary studies needed to reach statistical significance at the meta-analytic level. The second threshold, the *cluster size threshold (CST)*, which is used to correct for multiple comparisons, is computed through a series of computationally intensive Monte Carlo simulations of the null hypothesis. This CST reflects the cluster size needed to obtain statistical significance after correction for a false-discovery rate (FDR) of α = 0.05.

In later iterations of MKDA, *ensemble thresholding* is applied and involves a stepwise procedure of examining pairwise combinations of thresholds ranging from α = 0.05–0.0001. This process minimizes cluster size detection bias by capturing various ranges of values on both the MAST and the CST, which are inversely related. For example, a single MAST at α = 0.05 requires a large CST to obtain an FDR at α = 0.05 and therefore excludes small clusters with highly significant meta-analytic statistics. Inversely, a single MAST at α = 0.0001 involves a small CST to obtain an FDR at α = 0.0001 and therefore excludes large clusters with lower meta-analytic statistics that still reach statistical significance. Therefore, an ensemble of stepwise thresholds ranging from α = 0.05–0.0001 captures a more comprehensive and unbiased set of significant clusters of various sizes [11]. For a summary of major study design features of MKDA, as well as other types of meta-analyses, see Appendix A.

MKDA, as with other well-established CBMA methods such as activation likelihood estimation (ALE) [16] and signed differential mapping (SDM) [17], generates whole-brain meta-analytic maps from reported peak or center-of-mass coordinates [18]. However, it also differs in at least three important ways. First, MKDA treats opposing directions of activation reported by primary studies at overlapping brain regions as conflicting evidence and addresses this by computing difference scores at the meta-analytic level. Second, rather than relying on effect sizes, which are often inconsistently reported or thresholded in primary studies, MKDA utilizes weighted proportions [18]. Third, to mitigate cluster-size detection bias, MKDA can apply the ensemble thresholding procedure described above. Notably, although one study has found that SDM, which uses effect sizes when available, can enhance accuracy over traditional CBMA approaches [17], this study used a previous iteration of MKDA that did not employ ensemble thresholding. In addition, each of these CBMA approaches is limited by their reliance on published and thresholded coordinate data, which other techniques, such as shared repositories and consortia that use individual participant data (see Section 4), aim to address.

### 2.3. Applications and Findings

MKDA has been applied to the investigation of multiple psychiatric disorders (see Table 1). In an early application of MKDA, Etkin and Wager (2007) investigated post-traumatic stress disorder (PTSD; N = 19 studies; n = 175 participants) as well as social anxiety disorder (SAD; N = 11 studies; 73 participants) and specific phobia (N = 10 studies; 76 participants) [9]. In particular, these investigators found a pattern of fMRI hyperactivation across all three anxiety disorders, relative to healthy controls (HCs), in the amygdala and insula during negative emotional processing and fear-conditioning tasks, which reflected a common, transdiagnostic neural circuitry. In addition, they found disorder-specific activation patterns in participants diagnosed with PTSD, including hypoactivation during affective processing tasks in emotion regulation regions such as the dorsal anterior cingulate cortex (dACC) and ventromedial prefrontal cortex (vmPFC) [9].

Hamilton et al. (2012) later applied MKDA to investigate major depressive disorder (MDD) in adults (N = 38 studies; 1304 participants) [10]. These investigators found a robust pattern of hypoactivation in adults with MDD, relative to HCs, in emotion regulation regions such as the dorsolateral PFC (dlPFC) as well as hyperactivation in limbic regions such as the dorsal dACC, insula, and amygdala during negative emotional processing tasks [10]. These findings supported, in part, a frontolimbic model of depression [20,27], which involves impaired top-down inhibitory control between frontal and limbic regions, particularly the dlPFC and subgenual ACC (sgACC), during emotion regulation.

Subsequently, Miller et al. (2015) applied MKDA to investigate MDD in youth (N = 14 studies; 520 participants) [11]. This study found a robust pattern of hyperactivation in youth with MDD, relative to HCs, in a widely distributed range of cortical and limbic regions, including the dlPFC, sgACC, and insula, across a variety of affective and executive tasks. This study also expanded the MKDA toolkit to include ensemble thresholding and to facilitate quantitative, hierarchical comparisons of clinical and demographic groups [11]. A later study by Baten et al. (2023) used MKDA to conduct a direct, quantitative comparison of youth vs. adults with MDD [19]. This study found significant hyperactivation in youth with MDD, relative to adults with MDD, in the dlPFC and proposed top-down disengagement as a mechanism to explain this age-related difference in neural activation. It also conducted additional hierarchical comparisons to disentangle the effects of age and length of illness for each of the clusters that reached significance [19].

Miller (2018) explored the neural aberrations of adults with MDD relative to those of bipolar disorder (BD) and generalized anxiety disorder (GAD) [7]. These studies found subsets of neural regions that were *depression-specific* as well as regions that were *category-specific*, including *mood-specific* (i.e., MDD and BD only) or *distress-specific* (MDD and GAD only), or *transdiagnostic* (i.e., MDD, BD, and GAD). Collectively, these comparative analyses showed a complex set of findings that revealed partially overlapping neural signatures for each disorder [7].

In addition, Kaiser et al. (2015) used MKDA to analyze resting-state functional connectivity fMRI studies of MDD (N = 25 studies; n = 1074 participants) [20]. This meta-analysis found a pattern of hyperconnectivity within the default mode network (DMN), reflecting over-engagement of self-referential processing, as well as hypoconnectivity within the frontoparietal network (FPN), between the FPN and dorsal attention network (DAN), reflecting impairment in cognitive control of attention and emotion regulation. These findings provide a network-level model of neurocognitive dysfunction of depression that accounts for several of the neural and behavioral symptoms of MDD [20].

## 3. Meta-Analyses of Positron Emission Tomography Neuroimaging of Neuroinflammation

### 3.1. Overview

Neuroinflammation, which is characterized by an immune response and inflammation in the brain that can compromise function of the blood–brain barrier [28], is increasingly associated with a range of psychiatric and neurological diseases, such as depression [29] and neurodegeneration [30]. The development of techniques that can identify neuroinflammation has the potential to advance our understanding of its role in psychiatric and neurological disease and to provide new treatment approaches. Positron emission tomography imaging is particularly useful in visualizing and quantifying neuroinflammation, as it can use radiotracers to bind to specific molecular targets involved in inflammatory responses and provide a noninvasive, in vivo measurement across the whole-brain [24,31]. The application of meta-analyis to primary studies that investigate neuroinflammation via PET techniques in psychiatric and neurological diseases increases stastistical power and generalizabilty as well as our understanding of heterogeneity between individual primary studies.

### 3.2. Methodology

PET meta-analyses, similar to MKDA of fMRI studies, involve three phases. First, during *literature screening*, databases are searched according to preregistered inclusion criteria [11] and in accordance with PRISMA guidelines [12]. Typically, selected studies involve case–control comparisons of human studies using translocator protein (TSPO) tracers of neuroinflammation that target 18 kDa TSPO, such as [^11^C]-PK11195, [^11^C]-PBR28, or [^18^F]-DPA-714, and report a quantitative binding outcome such as binding potential (BP), distribution volume (Vt), or standardized uptake value ratio (SUVR). Second, during *data extraction*, independent coding teams obtain and organize relevant information from each study, including region-of-interest (ROI) labels and associated outcome data, which are typically reported as mean differences and standard deviations in binding measures for cases vs. controls, as well as other study characteristics. Third, *data analysis* is conducted by computing effect sizes (e.g., standardized mean difference [SMD], Hedges’ *g*), for each ROI according to a random-effects model. Additionally, secondary analyses are often performed to inspect heterogeneity, quality assessment, single-study sensitivity, and publication bias. Although capable of detecting neuroinflammatory markers, PET studies may be limited by relatively small sample sizes and tracer heterogeneity [24,25]

### 3.3. Applications and Findings

PET meta-analyses of neuroinflamation have been applied to the investigation of neuroinflammation across multiple neurological diseases (see Table 1). For example, in a meta-analysis of neuroinflammation in dementia, Pan et al. (2024) examined 12 primary studies of over 800 participants [24]. This study found elevated TSPO expression in dementia participants, relative to HCs, in the hippocampus but not in the anterior cingulate, parietal cortex, thalamus, or striatum. This study also found that cognitive impairment was associated with elevated TSPO in the prefrontal cortex and cingulate gyrus. This pattern of findings suggests region-specific mapping of neuroinflammation, particularly in the hippocampus, in dementia and that further research is needed to address variability in methodology across studies [24].

In another PET meta-analysis of neuroinflammation in Parkinson’s disease (PD), Zhang and Gao (2022) examined 15 primary studies of over 400 participants [25]. This meta-analysis found, in studies using first-generation ligands, elevated TSPO expression in PD participants, relative to HCs, across a range of brain regions, including those characteristically affected in PD such as the substantia nigra, midbrain, caudate, putamen, hippocampus, and cerebral cortex. However, in studies using second-generation ligands, PD participants, relative to HCs, exhibited elevated TSPO expression in the midbrain only. These findings suggest that neuroinflammation is not only present in PD but broadly distributed across many brain regions and that development and application of a variety of tracers with different sensitivities and specificities may be particularly important in detecting the longitudinal development of neuroinflammation in PD [25].

In addition, De Picker et al. (2023) conducted a transdiagnostic study of neuroinflammation of 156 case–control studies using PET imaging [26]. This meta-analysis found that across multiple disorders, including neurodegenerative disorders (e.g., Alzheimer’s disease [AD], PD), neuropsychiatric disorders (e.g., MDD, schizophrenia), traumatic brain injury, autoimmune, and other disorders, TSPO PET signal was significantly higher in cases vs. controls in cortical gray matter (SMD = 0.36) and that, by disorder, AD and neurodegenerative conditions show particular patterns of elevation in certain regions. These results suggest that neurological and neuropsychiatric disorders may exhibit both a transdiagnostic biomarker of neuroinflammation in cortical gray matter and a disease-specific neuroinflammatory profile [26].

## 4. Enhancing Neuroimaging Genetics Through Meta-Analysis (ENIGMA) Consortium Neuroimaging Protocols

### 4.1. Overview

The Enhancing Neuroimaging Genetics through Meta-Analysis (ENIGMA) Consortium [29,30] is a global collaborative research initiative that aims to advance our understanding of the neuroimaging and genetic markers of human disorders and traits by harmonizing neuroimaging protocols and increasing reproducibility. This individual participant data meta-analysis (IPD-MA) platform has a current size of over 50,000 processed MRI scans from 45 countries and has generated over 100 peer-reviewed publications. The ENIGMA Consortium is organized into 50 active working groups and 4 key research cores, including psychiatric and neurological disorders, developmental trajectories, genetics, and methodological innovations [32,33].

### 4.2. Methodology

The ENIGMA Consortium employs a harmonized, decentralized and federated analytic workflow. This approach enables participating sites to preprocess imaging data and then conduct statistical modeling with standardized linear models and control for covariates using harmonized protocols with tools such as Freesurfer, FSL, SPM, R, MATLAB, and Python. To conduct a centralized meta-analysis, summary statistics are then submitted to a coordinating center, where they are combined with other site-level data using random-effects inverse variance-weighted meta-analysis and meta-regression models. Although harmonization procedures are used, variability across sites and cohorts may still introduce limitations and biases [32,34].

### 4.3. Applications and Findings

The ENIGMA consortium has published multiple studies that have advanced our understanding of psychiatric disorders (see Table 1). In an application of the meta-analytic workflow of the ENIGMA MDD Working Group, Schmaal et al. (2016) [21] explored MRI subcortical volumes in a sample of 1728 individuals with MDD and 7199 HCs from 15 international cohorts. This study found a statistically significant reduction in hippocampal volume in participants with MDD relative to HCs, particularly in individuals with recurrent episodes and an early age of onset, which may reflect illness chronicity and stress-related neurotoxicity [21].

In another meta-analysis using the workflow of the ENIGMA Schizophrenia Working Group, Kelly et al. (2018) [22] examined DTI white matter microstructure in a sample of 1963 individuals with schizophrenia and 2359 healthy controls from 29 cohorts. This study found widespread reductions in fractional anisotropy (FA) across the brain as well as affected tracts such as the corpus callosum, superior longitudinal fasciculus, and internal capsule in participants with schizophrenia, reflecting diffuse white matter disruption in this disorder [22].

In the first large-scale fMRI meta-analysis of the ENIGMA-PGC PTSD Consortium, Zhu et al. (2023) [23] trained a classifier on resting-state fMRI data from 2495 participants and 20 sites to distinguish between individuals with PTSD and HCs. In particular, the highest-performing support vector machine (SVM) performance accuracy of up to 75% when distinguishing between participants with PTSD and those without trauma history after training using a denoising variational autoencoder (DVAE) to help reduce inter-site variability. This study demonstrated the feasibility of a large-scale, multi-site computational approach to objective classification of a psychiatric disorder using resting-state fMRI data [23].

## 5. Meta-Genome-Wide-Association Studies and Polygenic Risk Scores

### 5.1. Overview

Meta-analytic techniques have been widely used to combine GWASs to improve statistical power and the chance of detecting genetic variants with small effects [35]. PRS methods, which quantify the weighted summary of a person’s genetic risk for a particular trait or disease independent of environmental factors, have substantially added to our understanding of genetic risk of various diseases, including neuropsychiatric conditions, particularly those that are highly polygenic and multimorbid [36].

### 5.2. Methodology

Clumping and thresholding (C + T) is a common procedure used to compute PRSs from single nucleotide polymorphism (SNP) effect sizes from a GWAS [37,38]. *Clumping* (or linkage disequilibrium [LD] pruning) refers to the process of retaining only the most statistically significant SNP in a given region, to avoid double-counting highly correlated SNPs, by removing or “clumping” nearby, correlated SNPs. *Thresholding* involves retaining only SNPs with GWAS *p*-values below a chosen threshold, which is often chosen based on their predictive performance in a target sample. Following this process the PRS is calculated by summing the weighted risk alleles across the retained SNPs for each individual in the test dataset. In addition, non-traditional methods have been implemented using both Bayesian and frequentist penalized regressions [37,38]. Because meta-analyses provide more accurate estimates and yield a greater number of associated SNPs than individual studies, they represent an ideal basis for constructing PRSs [37,39]. However, PRS studies are limited in their proportion of trait variance explained and their generalizability across non-European ancestries [37].

### 5.3. Applications and Findings

Meta-GWAS and PRS studies have resulted in significant advancements in our understanding of multiple neuropsychiatric disorders (see Table 2). The identification of multi-gene-related risk through PRSs in neuropsychiatric disorders was initiated by a landmark study by the International Schizophrenia Consortium (ISC) in 2009 [40]. This Meta-GWAS included over 6800 cases and controls from multiple cohorts across Europe and the United States and used a PRS approach to predict case–control status of schizophrenia. Though no single SNP had a large impact, the cumulative effect of thousands of common SNPs was substantial and explained a significant proportion of risk for schizophrenia. Moreover, schizophrenia PRSs also predicted risk for bipolar disorder, which provided early evidence of shared genetic architecture between these two psychiatric disorders. Importantly, this landmark study established PRSs as a key method in psychiatric genetics and provided early empirical evidence that psychiatric disorders are highly polygenic and share common genetic variation [40].

Subsequently, the ISC merged with other early GWAS efforts to form the Psychiatric Genomics Consortium (PGC) [39], which has become the largest international collaborative effort in psychiatric genetics, with pooled data from over 1 million participants and 800 research groups from over 40 countries [44]. Their use of PRSs derived from meta-analytic GWASs has led to the identification of predictive models and genetic loci in multiple psychiatric disorders, including schizophrenia [45], MDD [39], bipolar disorder [46], and PTSD [47] as well as cross-disorder analyses that demonstrate the genetic relations between psychiatric disorders [48]. Collectively, these studies have fundamentally shifted the field of psychiatric genetics from low-replication candidate-gene studies to robust, large-scale GWAS meta-analyses and advanced our understanding of the genetic basis of psychiatric disorders as highly polygenic, pleiotropic, disorders that are often best represented by spectrum models [44].

For example, Grove et al. (2019) conducted a large-scale Meta-GWAS by combining a Danish population-based sample of 13,076 individuals with autism spectrum disorder (ASD) and 22,664 case controls with the previously published PGC data of 5305 cases and 5305 controls for a larger meta-analytically derived sample [41]. These investigators found that the highest decile of ASD PRS was associated with a significantly greater chance of ASD diagnosis (OR: 2.80; 95% CI: 2.53–3.10). They also identified 5 genome loci linked to ASD and demonstrated that some of the ASD genes were linked to schizophrenia, depression, educational attainment, and intellectual function. They further demonstrated a differential genetic loading for ASD among those with and without intellectual disability; those without intellectual disability had higher contributions from common variants, suggesting a stronger polygenic architecture for this ASD phenotype. Those with ASD and intellectual disability had weaker common variant effects, leading the authors to suggest that such clinical states may be related to rare mutations and could inform the development of clinical subtypes [41].

Other investigators have also employed Meta-GWASs and PRSs to examine neuropsychiatric disorders. For example, in their meta-analysis, Kubota et al. (2025) examined the predictive value of PRSs on epilepsy risk in 11 primary studies [42]. These investigators demonstrated that PRSs are predictive of epilepsy overall, with a stronger association for generalized versus focal epilepsy. They also examined the odds ratio for developing epilepsy while examining a risk gradient as PRSs increase. Specifically, risk estimates increased with PRS percentiles. The risk for developing generalized epilepsy among the top 20% of the PRS distribution was 2.18 (95% CI: 1.91–2.48), 2.65 (95% CI: 2.07–3.39) in the top 5%, and 4.62 (95% CI: 3.45–6.20) in the top 0.5%. PRSs were also predictive of focal epilepsy, but only among the top 5 and top 0.5 percent of the PRS distribution (OR: 1.40; 95% CI: 1.28–1.52 and OR: 1.69; 95% CI: 1.27–2.24, respectively). In other words, epilepsy risk increases as genetic loading increases, but the predictive value of PRSs may be highest among those with more extreme genetic loadings and more so for generalized relative to focal cases [42].

Green et al. (2022) similarly examined the predictive value of PRSs in attention-deficit/hyperactivity disorder (ADHD) [43]. In particular, they analyzed 16 primary studies that examined risk of ADHD among children and demonstrated that higher PRSs are associated with increased risk of ADHD diagnosis (OR: 1.37; CI not reported) and greater ADHD symptom severity (β = 0.06; *p* < 0.001) as well as psychiatric comorbidities, including anxiety (OR: 1.16) and irritability and emotional dysregulation (OR: 1.14). Notably, this study represents a sophisticated application of meta-analytic techniques to PRS methodology by examining both binary (diagnosis) and continuous (symptom severity) outcomes as well as comorbid psychiatric symptoms, which provides a nuanced perspective on ADHD genetic liability [43].

## 6. Conclusions

Recent advancements in neuroimaging and genetics have generated a rapid proliferation of primary studies in these fields, leading to the development and application of meta-analytic methods, which have contributed substantially to our understanding of psychiatric and neurological disorders. In particular, the development of meta-analytic methods in neuroimaging has contributed to the refinement of robust neural models for depression and other psychiatric disorders and an understanding of transdiagnostic and disease-specific biomarkers of inflammation that may inform clinical interventions as well as the formation of international consortia to enhance generalizability of findings and accelerate clinical translation. In addition, the application of meta-analytic methods in genetics has led to the discovery of numerous risk variants, enabled sophisticated risk prediction models, and underscored the polygenic and pleiotropic architecture of psychiatric and neurological disorders. Future research should continue to develop techniques for harmonizing multimodal data types, particularly neuroimaging and multi-omic data, to advance more comprehensive models of brain function and pathology that cannot be captured by any single modality. It should also pursue both biomarker- and mechanism-driven approaches to strengthen predictive models of disease and to build new theoretical frameworks that guide future research and yield new discoveries.

Finally, researchers should leverage findings to inform the development of precision treatments and to enhance the biological accuracy and relevance of diagnostic systems.

## Figures and Tables

**Table 1:** Examples of neuroimaging meta-analyses of psychiatric and neurological disorders.

Imaging Technique	Study	Diagnosis	Primary Studies	Findings
fMRI activation & metabolic PET	Etkin & Wager (2007) [9]	PTSD, social anxiety, & specific phobia	20 fMRI & PET studies	Hyperactivation across disorders in amygdala & insula during negative emotional processing & fear conditioning; hypoactivation in PTSD in dACC & vmPFC during affective processing; findings support transdiagnostic and disorder-specific neural circuitry
fMRI task-based & resting-state & metabolic & perfusion PET	Hamilton et al. (2012) [10]	MDD	49 fMRI & PET studies	Hypoactivation in MDD in emotion regulation regions (e.g., dlPFC & dACC) during cognitive control & reappraisal & hyperactivation in limbic regions (e.g., rACC, insula, amygdala) during negative emotional processing; findings support frontolimbic model of MDD
fMRI activation	Miller et al. (2015) [11]	MDD in youth	14 fMRI studies	Hyperactivation in multiple cortical & limbic regions (e.g., dlPFC, sgACC, insula) across variety of affective & executive tasks; findings partially diverge from those of adults with MDD
fMRI activation	Baten et al. (2023) [19]	MDD in youth vs. adults	135 fMRI studies	Hyperactivation in multiple cortical & basal ganglia regions, particularly the dlPFC, that were differentially attributable to age & length of illness; finding support developmental differences in MDD
fMRI activation	Miller (2018) [7]	MDD, BD, & GAD	96 fMRI studies	Hyperactivation in multiple cortical & limbic regions across variety of affective & executive tasks; findings support transdiagnostic, category-specific, & disorder-specific effects
fMRI functional connectivity	Kaiser et al. (2015) [20]	MDD	25 fMRI studies	Hyperconnectivity within the DMN; hypoconnectivity within the FPN & between the FPN & DAN
MRI	Schmaal et al. (2016) [21]	MDD	15 MRI cohorts	Reduced hippocampal volume in MDD
DTI	Kelly et al. (2018) [22]	Schizophrenia	29 DTI cohorts	Reduced fractional anisotropy across brain and in multiple tracts (e.g., corpus callosum, superior longitudinal fasciculus, internal capsule) in schizophrenia)
Resting-state fMRI	Zhu et al. (2023) [23]	PTSD	20 fMRI sites	SVM distinguished between PTSD and HC participants with up to 75% accuracy
TSPO PET	Pan et al., 2024 [24]	Dementia (AD, MCI, & others)	12 PET studies	Elevated TSPO in dementia in hippocampus
TSPO PET	Zhang & Gao, 2022 [25]	Parkinson’s disease	15 PET studies	Elevated TSPO in PD in multiple regions, including midbrain; findings differ by ligand & tracer generation
TSPO PET	De Picker et al., 2023 [26]	Transdiagnostic (11 illness categories)	156 PET studies	Elevated TSPO across all included disorders in cortical grey matter; elevated TSPO by disorder in particular regions

Abbreviations: BD: bipolar disorder; dACC: dorsal anterior cingulate cortex; DAN: dorsal attention network; dlPFC: dorsolateral prefrontal cortex; DMN: default mode network; DTI: diffusion tensor imaging; fMRI: functional magnetic resonance imaging; FPN: frontoparietal network; GAD: generalized anxiety disorder; HC: healthy control; MDD: major depressive disorder; MRI: magnetic resonance imaging; PD: Parkinson’s disease; PET: positron emission tomography; PTSD: post-traumatic stress disorder; rACC: rostral anterior cingulate cortex; sgACC: subgenual anterior cingulate cortex; SVM: support vector machine; TSPO: translocator protein; vmPFC: ventromedial prefrontal cortex.

**Table 2:** Examples of Meta-Genome-Wide-Association Studies and meta-analyses of polygenic risk scores of psychiatric and neurological disorders.

Genetic Technique	Study	Diagnosis	Data Source	Findings
Meta-GWAS & PRS	International Schizophrenia Consortium 2009 [40]	Schizophrenia	Consortium with over 6800 cases & controls	Risk model explained significant proportion of risk for schizophrenia; demonstrated shared genetic risk between schizophrenia & bipolar disorder
Meta-GWAS & PRS	Grove et al. (2019) [41]	Autism spectrum disorder	Registries with over 45,000 cases & controls	Identified several genetic loci that reached genome-wide significance for ASD; demonstrated differential genetic loadings for ASD phenotypes with and without intellectual disability
Meta-GWAS & PRS	Kubota et al. (2025) [42]	Epilepsy	11 primary studies	Risk model predicted significant proportion of risk for epilepsy, particularly for those with more extreme genetic loadings and for generalized epilepsy
Meta-GWAS & PRS	Green et al. (2022) [43]	Attention-deficit/hyperactivity disorder	16 primary studies	Higher PRSs associated with increased risk of ADHD diagnosis & symptom severity as well as psychiatric comorbidities

Abbreviations: ADHD: attention-deficit/hyperactivity disorder; ASD: autism spectrum disorder; GWAS: Genome-Wide Association Study; PRS: polygenic risk score.

## Data Availability

No new data were created or analyzed in this study. Data sharing is not applicable to this article.

## Abbreviations

MKDA	multilevel kernel density analysis
fMRI	functional magnetic resonance imaging
PET	positron emission tomography
ENIGMA	Enhancing Neuroimaging Genetics through Meta-Analysis
GWAS	genome-wide association study
PRS	polygenic risk score
CBMA	coordinate-based meta-analysis
MAST	meta-analytic threshold
CST	cluster size threshold
FDR	false-discovery rate
ALE	activation likelihood estimation
SDM	signed differential mapping
PTSD	post-traumatic stress disorder
SAD	social anxiety disorder
HC	healthy control
dACC	dorsal anterior cingulate cortex
vmPFC	ventromedial prefrontal cortex
MDD	major depressive disorder
dlPFC	dorsolateral prefrontal cortex
sgACC	subgenual anterior cingulate cortex
BD	bipolar disorder
GAD	generalized anxiety disorder
DMN	default mode network
FPN	frontoparietal network
DAN	dorsal attention network
DTI	diffusion tensor imaging
MRI	magnetic resonance imaging
PD	panic disorder
rACC	rostral anterior cingulate cortex
SVM	support vector machine
TSPO	translocator protein
BP	binding potential
Vt	distribution volume
SUVR	standardized uptake value ratio
ROI	region of interest
SMD	standardized mean difference
AD	Alzheimer’s disease
IPD-MA	individualized participant data meta-analysis
FA	fractional anisotropy
DVAE	denoising variational autoencoder
C + T	clumping and thresholding
SNP	single nucleotide polymorphism
LD	linkage disequilibrium
ISC	International Schizophrenia Consortium
PGC	Psychiatric Genomics Consortium
ASD	autism spectrum disorder
ADHD	attention-deficit/hyperactivity disorder

## Appendix A

**Table A1:** Best practices for MKDA, PET neuroinflammation, ENIGMA, and meta-GWAS studies.

Item Number	Study Design Feature	Description
1	PROSPERO pre-registration	Pre-registration of study design and protocol
2	PRISMA checklist	Standardized set of items to report to ensure transparency, completeness, and reproducibility
3	Inclusion criteria	Predefined inclusion criteria used to screen literature
4	Double screening	Duplicate literature screening by independent teams to ensure completeness and accuracy
5	Monte Carlo simulations *	Compute cluster sizes needed to achieve correction for multiple comparisons and false discovery rates
6	Ensemble thresholding *	Stepwise examination of statistical thresholds to minimize cluster size detection bias
7	Risk of bias assessment	Conduct quality assessment of primary studies
8	Leave-one-out analysis	Compute robustness to single study removal
9	Fail-safe N analysis	Determine robustness to publication bias
10	File sharing	Sharing of tracker files and analysis script to promote reproducibility

* where applicable, such as MKDA.

## References

[1] Simard M.-A., Kozlowski D., Segal J., Messer M., Ocay D.D., Saari T., Ferland C.E., Larivière V. (2025). Trends in brain research: A bibliometric analysis. Can. J. Neurol. Sci..

[2] Yeung A.W.K., Goto T.K., Leung W.K. (2017). A bibliometric review of research trends in neuroimaging. Curr. Sci..

[3] Gurevitch J., Koricheva J., Nakagawa S., Stewart G. (2018). Meta-analysis and the science of research synthesis. Nature.

[4] Wager T.D., Lindquist M., Kaplan L. (2009). Meta-analysis of functional neuroimaging data: Current and future directions. Soc. Cogn. Affect. Neurosci..

[5] Button K.S., Ioannidis J.P.A., Mokrysz C., Nosek B.A., Flint J., Robinson E.S.J., Munafò M.R. (2013). Power failure: Why small sample size undermines the reliability of neuroscience. Nat. Rev. Neurosci..

[6] Eklund A., Nichols T.E., Poline J.B. (2016). Cluster failure: Why fMRI inferences for spatial extent have inflated false-positive rates. Proc. Natl. Acad. Sci. USA.

[7] Miller C.H. (2018). Disorder-Specific and Transdiagnostic Functional Neuroimaging Abnormalities in Major Depressive Disorder. Ph.D. Thesis.

[8] Wager T.D., Lindquist M., Kaplan L. (2009). Evaluating the consistency and specificity of neuroimaging data using meta-analysis: The MKDA framework. NeuroImage.

[9] Etkin A., Wager T.D. (2007). Functional neuroimaging of anxiety: A meta-analysis of emotional processing in PTSD, social anxiety disorder, and specific phobia. Am. J. Psychiatry.

[10] Hamilton J.P., Etkin A., Furman D.J., Lemus M.G., Johnson R.F., Gotlib I.H. (2012). Functional neuroimaging of major depressive disorder: A meta-analysis and new integration of baseline activation and neural response data. Am. J. Psychiatry.

[11] Miller C.H., Hamilton J.P., Sacchet M.D., Gotlib I.H. (2015). Functional neuroimaging of major depressive disorder in youth: A meta-analysis. JAMA Psychiatry.

[12] Moher D., Liberati A., Tetzlaff J., Altman D.G., PRISMA Group (2009). Preferred reporting items for systematic reviews and meta-analyses: The PRISMA statement. PLoS Med..

[13] Booth A., Clarke M., Dooley G., Ghersi D., Moher D., Petticrew M., Stewart L. (2012). The nuts and bolts of PROSPERO: An international prospective register of systematic reviews. Syst. Rev..

[14] Talairach J., Tournoux P. (1988). Co-Planar Stereotaxic Atlas of the Human Brain: 3-Dimensional Proportional System—An Approach to Cerebral Imaging.

[15] Evans A.C., Collins D.L., Neelin P., Peters T.M., White T.D. (1993). 3D statistical neuroanatomical models from 305 MRI volumes. Proceedings of the 1993 IEEE Conference Record Nuclear Science Symposium and Medical Imaging Conference.

[16] Eickhoff S.B., Laird A.R., Fox P.T., Lancaster J.L., Fox P.M. (2011). Activation likelihood estimation meta-analysis revisited. NeuroImage.

[17] Radua J., Mataix-Cols D. (2012). A new meta-analytic method for neuroimaging studies that combines reported peak coordinates and statistical parametric maps. Eur. Psychiatry.

[18] Müller V.I., Cieslik E.C., Laird A.R., Fox P.T., Radua J., Mataix-Cols D., Tench C.R., Yarkoni T., Nichols T.E., Turkeltaub P.E. (2018). Ten simple rules for neuroimaging meta-analysis. Neurosci. Biobehav. Rev..

[19] Baten C., Klassen A.M., Shepherd J.H., Zamora G., Pritchard E., Saravia S., Ali z., Zamora G., Jordan J., Kahlon S.K. Effects of developmental age and length of illness in major depressive disorder: A meta-analysis of functional magnetic resonance imaging studies. Proceedings of the Society of Biological Psychiatry Annual Meeting.

[20] Kaiser R.H., Andrews-Hanna J.R., Wager T.D., Pizzagalli D.A. (2015). Large-Scale Network Dysfunction in Major Depressive Disorder: A Meta-analysis of Resting-State Functional Connectivity. JAMA Psychiatry.

[21] Schmaal L., Veltman D.J., Van Erp T.G.M., Sämann P.G., Frodl T., Jahanshad N., Loehrer E., Tiemeier H., Hofman A., Niessen W.J. (2016). Subcortical brain alterations in major depressive disorder: Findings from the ENIGMA Major Depressive Disorder Working Group. Mol. Psychiatry.

[22] Kelly S., Jahanshad N., Zalesky A., Kochunov P., Agartz I., Alloza C., Andreassen O.A., Arango C., Banaj N., Bouix S. (2018). Widespread white matter microstructural differences in schizophrenia across 4322 individuals: Results from the ENIGMA Schizophrenia DTI Working Group. Mol. Psychiatry.

[23] Zhu X., Kim Y., Ravid O., He X., Suarez-Jimenez B., Zilcha-Mano S., Lazarov A., Lee S., Abdallah C.G., Angstadt M. (2023). Neuroimaging-based classification of PTSD using data-driven computational approaches: A multisite big data study from the ENIGMA-PGC PTSD Consortium. NeuroImage.

[24] Pan J., Hu J., Meng D., Chen L., Wei X. (2024). Neuroinflammation in dementia: A meta-analysis of PET imaging studies. Medicine.

[25] Zhang P.-F., Gao F. (2022). Neuroinflammation in Parkinson’s disease: A meta-analysis of PET imaging studies. J. Neurol..

[26] De Picker L.J., Morrens M., Branchi I., Haarman B.C., Terada T., Kang M.S., Boche D., Tremblay M.-E., Leroy C., Bottlaender M. (2023). TSPO PET brain inflammation imaging: A transdiagnostic systematic review and meta-analysis of 156 case-control studies. Brain Behav. Immun..

[27] Mayberg H.S. (1997). Limbic-cortical dysregulation: A proposed model of depression. J. Neuropsychiatry Clin. Neurosci..

[28] Chaudhary S., Chao H., Agarwal K., Gupta S., Li C. (2025). Imaging Markers of Neuroinflammation in Aging and Alzheimer’s Disease and Related Dementia: A Comprehensive Review. Neurosci. Biobehav. Rev..

[29] Beurel E., Toups M., Nemeroff C.B. (2020). The bidirectional relationship of depression and inflammation: Double trouble. Neuron.

[30] Kwon H.S., Koh S.H. (2020). Neuroinflammation in neurodegenerative disorders: The role of microglia and astrocytes. Neurodegener..

[31] Canário N., Jorge L., Martins R., Santana I., Castelo-Branco M. (2022). Dual PET-fMRI reveals a link between neuroinflammation, amyloid binding and compensatory task-related brain activity in Alzheimer’s disease. Commun. Biol..

[32] Thompson P.M., Stein J.L., Medland S.E., Hibar D.P., Vasquez A.A., Renteria M.E., Toro R., Jahanshad N., Schumann G., Franke B. (2014). The ENIGMA Consortium: Large-scale collaborative analyses of neuroimaging and genetic data. Brain Imaging Behav..

[33] ENIGMA Consortium ENIGMA: Enhancing NeuroImaging Genetics Through Meta-Analysis. https://enigma.ini.usc.edu/.

[34] Schmaal L., Pozzi E., Ho T.C., van Velzen L.S., Veer I.M., Opel N., Van Someren E.J.W., Han L.K.M., Aftanas L., Aleman A. (2020). ENIGMA MDD: Seven years of global neuroimaging studies of major depression through worldwide data sharing. Psychiatry.

[35] Zeggini E., Ioannidis J.P. (2009). Meta-analysis in genome-wide association studies. Pharmacogenomics.

[36] Andreoli L., Peeters H., Van Steen K., Dierickx K. (2025). Polygenic risk scores in the clinic: A systematic review of stakeholders’ perspectives, attitudes, and experiences. Eur. J. Hum. Genet..

[37] Choi S.W., Mak T.S., O’Reilly P.F. (2020). Tutorial: A guide to performing polygenic risk score analyses. Nat. Rev. Genet..

[38] Ndong Sima C.A.A., Step K., Swart Y., Schurz H., Uren C., Möller M. (2024). Methodologies underpinning polygenic risk scores estimation: A comprehensive overview. Hum. Genet..

[39] Wray N.R., Ripke S., Mattheisen M., Trzaskowski M., Byrne E.M., Abdellaoui A., Adams M.J., Agerbo E., Air T.M., Andlauer T.M.F. (2018). Genome-wide association analyses identify 44 risk variants and refine the genetic architecture of major depression. Nat. Genet..

[40] International Schizophrenia Consortium (2009). Common polygenic variation contributes to risk of schizophrenia and bipolar disorder. Nature.

[41] Grove J., Ripke S., Als T.D., Mattheisen M., Walters R.K., Won H., Pallesen J., Agerbo E., Andreassen O.A., Anney R. (2019). Identification of common genetic risk variants for autism spectrum disorder. Nat. Genet..

[42] Kubota T., Ngadimon I.W., Ohseto H., Viswanathan S., Ravat P., Acharya M.K., Kuroda N., Konomatsu K., Obara T., Jin K. (2025). Predictive value of the polygenic risk score for developing epilepsy: A systematic review and meta-analysis. Epilepsy Behav..

[43] Green A., Baroud E., DiSalvo M., Faraone S.V., Biederman J. (2022). Examining the impact of ADHD polygenic risk scores on ADHD and associated outcomes: A systematic review and meta-analysis. J. Psychiatr. Res..

[44] Sullivan P.F., Agrawal A., Bulik C.M., Andreassen O.A., Børglum A.D., Breen G., Cichon S., Edenberg H.J., Faraone S.V., Gelernter J. (2018). Psychiatric genomics: An update and an agenda. Am. J. Psychiatry.

[45] Schizophrenia Working Group of the Psychiatric Genomics Consortium (2014). Biological insights from 108 schizophrenia-associated genetic loci. Nature.

[46] Mullins N., Forstner A.J., O’connell K.S., Coombes B., Coleman J.R.I., Qiao Z., Als T.D., Bigdeli T.B., Børte S., Bryois J. (2021). Genome-wide association study of more than 40,000 bipolar disorder cases provides new insights into the underlying biology. Nat. Genet..

[47] Nievergelt C.M., Maihofer A.X., Klengel T., Atkinson E.G., Chen C.-Y., Choi K.W., Coleman J.R.I., Dalvie S., Duncan L.E., Gelernter J. (2019). International meta-analysis of PTSD genome-wide association studies identifies sex- and ancestry-specific genetic risk loci. Nat. Commun..

[48] Cross-Disorder Group of the Psychiatric Genomics Consortium (2013). Genetic relationship between five psychiatric disorders estimated from genome-wide SNPs. Nat. Genet..

